# Zebra stripes: the questions raised by the answers

**DOI:** 10.1111/brv.70063

**Published:** 2025-07-31

**Authors:** Hamish M. Ireland, Graeme D. Ruxton

**Affiliations:** ^1^ Department of Radiology Royal Infirmary of Edinburgh and University of Edinburgh 51, Little France Crescent Edinburgh EH16 4SA UK; ^2^ School of Biology, University of St Andrews St Andrews KY16 9ST UK

**Keywords:** equids, lion predation, tabanids, crypsis, thermoregulation, interspecific signalling, mixed‐species groups

## Abstract

Multiple hypotheses have been suggested to explain why the three zebra species (*Equus quagga*, *E. grevyi* and *E. zebra*) are striped. We review how well these theories explain the nature (rather than simply the existence) of the stripes. Specifically, we explore how well different theories explain (*i*) the form of zebra stripes (especially on different body parts), (*ii*) stripe variation between zebra populations and among species, and (*iii*) the lack of striping in other equids or other large mammalian herbivores. The main hypotheses discussed during the last decade are the deterrence of biting flies, thermoregulation through stripe‐generated air movement, and three anti‐predation hypotheses: crypsis to avoid detection; dazzle colouration to confuse pursuers; and interspecies signalling to encourage protective mixed‐species herding. Our evaluation suggests that these theories struggle to explain all aspects of variation in striping. For each theory we identify where through logical reasoning or empirical data, the theory is unable to account for an aspect of variation, or whether information is currently lacking. In the latter case we offer concrete suggestions for the types of empirical study that would be most useful. Deterrence of biting flies is the theory that currently has strongest empirical support, but this theory alone struggles to explain why striping occurs so strongly in zebra but not in other African mammals, and the distribution of stripes across the body. These aspects can be explained by the interspecies signalling theory, but this theory has not been empirically evaluated. We suggest how future studies could best utilise our framework to close the most pressing knowledge gaps in our understanding of this iconic example of animal colouration.

## I. INTRODUCTION

**Fig. 1:**
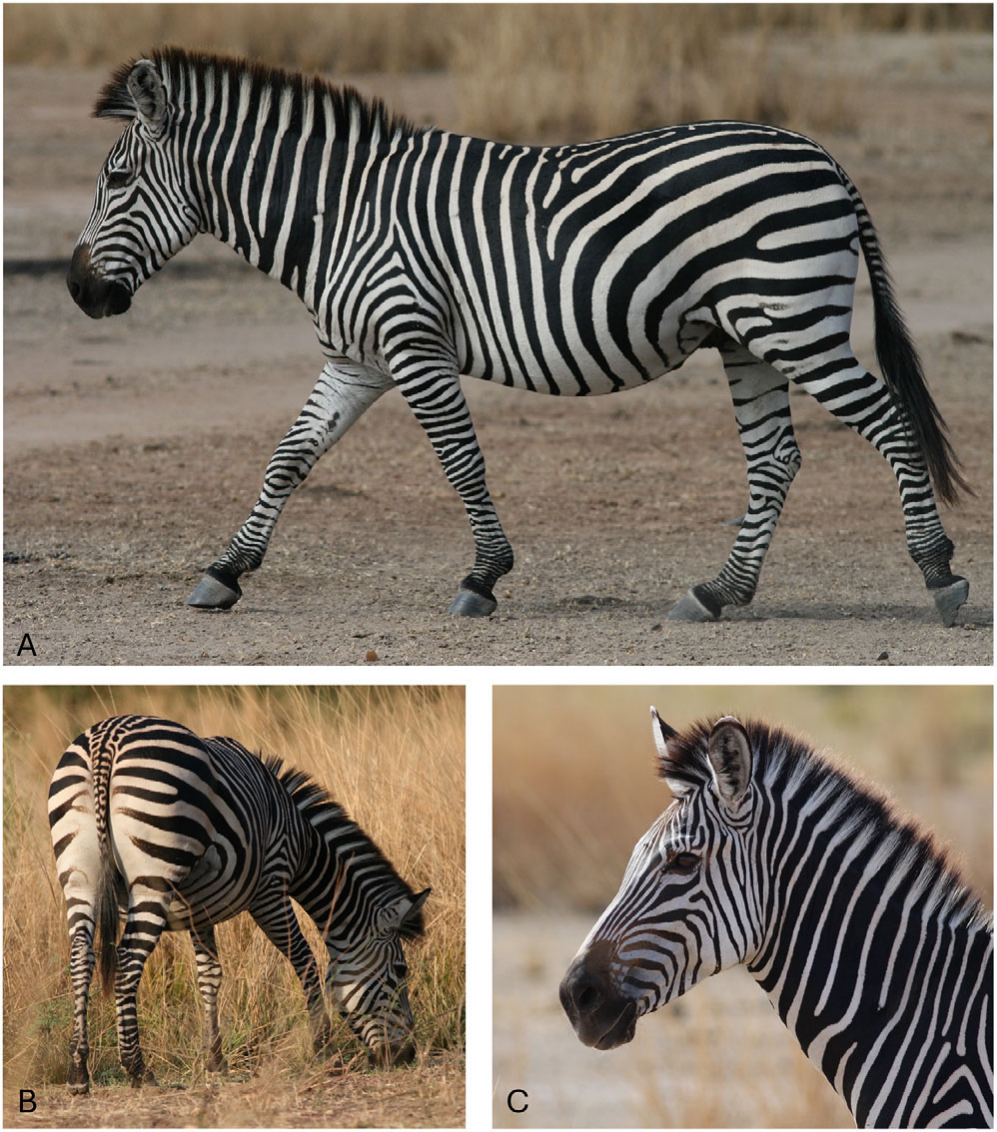
(A) Plains zebra (subspecies *Equus quagga crawshayi*) in Zambia (upper range of north–south striping gradient). Crawshay's zebra subspecies has narrower stripes than Grant's zebra (subspecies from Tanzania/Kenya). Note variation in stripe ratio with high black to white stripe ratio in neck and equal stripe ratio on rear flank. Note ratio alters from top of flank to low flank/belly which could provide countershading. (B) Plains zebra (*Equus quagga crawshayi*) showing whiter hindquarters/rump and striped tail. (C) Plains zebra (*Equus quagga crawshayi*). Note wide black and thin white stripes on neck which are conspicuous at short distance and less conspicuous at greater distance. Note wide white stripes on head creating a lighter average colour which will increase conspicuousness of head, particularly in low light. Note tall striped mane with black line at edge. All photographs by H. and A. Ireland.

The longstanding search for the elusive function or functions of zebra stripes (*Equus quagga*, *E. grevyi* and *E. zebra*) has led to multiple hypotheses. These are summarised elsewhere (Ruxton, 2002; Egri *et al*., 2012; Caro, 2016; Horváth *et al*., 2018). In the last decade, the main hypotheses discussed in the literature are the deterrence of biting flies, thermoregulation through stripe‐generated air movement and three hypotheses related to reducing predation: crypsis to avoid detection; ‘dazzle’ colouration to hinder tracking by pursuit predators; and interspecies signalling to encourage protective mixed‐species herding (Caro *et al*., 2014; How & Zanker, 2014; Larison *et al*., 2015; Melin *et al*., 2016; Ireland & Ruxton, 2017; Cobb & Cobb, 2019). The references above explore the strengths of these functions to contribute to selection for a striped appearance in zebra. Here we offer a fresh perspective – asking not how well these functions can help explain the existence of stripes in zebra, but how well each can explain aspects of the form and variation in stripes actually seen in zebra. We assess each hypothesis using a common list of zebra stripe features. This is to ensure diverse aspects of the zebra's unusual stripes (Fig. 1) are assessed methodically for each hypothesis. Our discussion is mainly centred around the plains zebra (*Equus quagga*), the commonest species, with additional comments regarding mountain zebra (*E. zebra*) and Grevy's zebra (*E. grevyi*) for each hypothesis. Our hope is that refocussing discussion from ‘Why do zebras have stripes?’ to ‘How can we explain the nature and variation of striping seen in zebra?’ will help open up new lines of investigation and enrich our understanding of this iconic example of animal colouration.

Table 1 provides a list of fundamental questions considered for each hypothesis discussed herein.

**Table 1:** Stripe feature questions considered for each hypothesis.

Question number	Stripe feature question
1	Is a striping pattern (as opposed to another form of contrasting pattern – such as spots or patches) compatible with the hypothesis?
2	Are black and white the most compatible colours, as opposed to other colours or shades?
3	Are stripe contrast and sharpness compatible?
4	Are black stripe widths for each body area compatible?
5	Is the ratio of black stripe width to white stripe width compatible? The width of the white stripes compared to black stripes should have a significant effect on overall conspicuousness (Iizuka *et al*., 2021).
6	Is stripe angle, i.e. relative to horizontal, compatible?
7	Is stripe upper body stripe distribution compatible?
8	Is head and neck stripe distribution compatible?
9	Is belly striping compatible?
10	Is leg striping compatible?
11	Is hindquarter striping compatible?
12	Is the striped mane compatible?
13	Is the striped tail compatible?
14	Is zebra behaviour compatible?
15	Does the hypothesis explain why only zebra are striped among sympatric African herbivores?
16	Does the hypothesis explain why equids elsewhere are not striped?
17	Does the hypothesis explain the gradient of increasing stripe vividness from south to north?
18	Does the hypothesis fit for mountain zebra and Grevy's zebra (as well as the plains zebra)?
19	Does the hypothesis have supporting data?

## II. BITING FLIES

### (1) Basis of hypothesis and evidence

There is extensive evidence showing that several species of biting flies prefer not to alight on striped surfaces, and prefer to land on dark shiny surfaces (e.g. How *et al*., 2020). This attraction to dark surfaces may be because most hosts are dark, or dark surfaces are easier to detect, but could also be to aid fly crypsis and to provide warmth for a rapid escape flight (Horváth *et al*., 2020).

The term ‘biting flies’ has been used because it has been unclear which fly species zebra stripes were capable of repelling. Initially tsetse flies (Diptera: Glossinidae) were suggested by Waage (1981) who found that striped models attracted fewer tsetse flies. However, the favoured hosts of savannah tsetse (*Glossina morsitans*, *G. austeni*, *G. auscipleuris* and *G. longipennis*) subsequently have been found to be the Suidae (e.g. warthog *Phacochoerus africanus*) (Clausen *et al*., 1998). For *G. swynnertoni* and *G. pallidipes* in Tanzania, the main hosts were warthog, buffalo *Syncerus caffer* and giraffe *Giraffa camelopardalis* and the following species were avoided despite being common: blue wildebeest *Connochaetes taurinus*, plains zebra, impala *Aepyceros melampus* and Thomson's gazelle *Eudorcas thomsonii* (Auty *et al*., 2016). As the avoided mammals are largely not striped, this suggests that factors other than stripes are likely involved in host preference for tsetse.

More recently, papers have focussed on horse flies/tabanids (Diptera: Tabanidae) and also on the much smaller stable flies/*Stomoxys* spp. (Diptera: Muscidae) (Egri *et al*., 2012; Caro *et al*., 2019, 2023; How *et al*., 2020). These fly families have less need for blood meals compared to tsetse (glossinids). In glossinids, both sexes are entirely haematophagous, however in tabanids, males and females feed from flowers and only the female requires a blood meal before laying eggs; stable flies also feed from flowers but both sexes require a blood meal for reproduction (Ballard & Waage, 1988; Gibson & Torr, 1999; Taylor & Berkebile, 2008).

### (2) Host location

Biting diptera such as glossinids (tsetse), tabanids (horseflies) and *Stomoxys* spp. (stable flies) are only active during the day and locate hosts at distance using olfactory cues; they fly upwind in the odour plume (Gibson & Torr, 1999) and then at short range (e.g. 15 m for tabanids) switch to visual cues (Phelps & Vale, 1976). Tabanids, *Stomoxys* spp. and likely glossinids, can also visually detect and are attracted to linearly polarised light (positive polarotaxis) which can help them to detect water and also to differentiate dark sunlit host animals, which reflect polarised light, from dark background patches (Blake *et al*., 2023; Horváth, 2024). Visual cues are used for close‐range localisation and then after circling around the target, visual and odour cues are used to choose the alighting area (Gibson & Torr, 1999). Each fly species favours specific areas on the body or legs of the host (Waage & Davies, 1986; Phelps & Holloway, 1990). Different visual cues can be important for attraction as opposed to landing: for glossinids and tabanids, blue and black are attractive on approach but black is favoured for landing (Green, 1994; Gibson & Torr, 1999). Shape and mobility are also relevant with a mobile horizontal rectangle being most attractive in comparison to a suite of other tested options (Phelps & Vale, 1976; Gibson & Torr, 1999). This preferred target might be similar to a hosts' shape, size and mobility and black may also be attractive due to the potential for fly crypsis and warmth for escape flight (Horváth *et al*., 2020).

### (3) Fly experiments

Experiments have shown that various biting flies avoid all‐white and contrasting striped (horizontal and vertical), spotty and checkered surfaces (Blaho *et al*., 2012; Egri *et al*., 2012; How *et al*., 2020; Sasaki *et al*., 2020; Caro *et al*., 2023), most recently mainly focussing on tabanids. In his book *Zebra Stripes*, Caro (2016) tried to reproduce the results of several previous fly experiments. For glossinids, in Tanzania, striped biconical and cloth traps did not reduce fly capture compared to black or brown traps but few flies were caught overall, and were mainly *G. morsitans* (which are normally attracted to movement). Caro then used himself as a lure by walking in different patterned suits; black and horizontal stripes attracted more glossinids than non‐striped. When walking while wearing pelts, there was no difference between plains zebra and blue wildebeest pelts, and when driving with pelt lures, more flies landed on a zebra pelt compared to a wildebeest pelt.

Caro then studied tabanids using canopy traps (Mullens, 2018), fewer tabanids were caught with a shiny striped ball lure than the usual shiny black lure. Using a canopy trap with pelts as a lure, zebra pelts were slightly (but not significantly) less attractive than wildebeest pelts. Following his experiments and comparisons, Caro (2016) tentatively suggested that stripes might be more of a deterrent for tabanids than glossinids.

Caro also compared multiple fly‐bothering indices, including tail‐swishing, between striped and non‐striped equids at Tierpark zoo in Berlin and found no effect of striping. In Tanzania, he again found no benefit of stripes: zebras appeared more bothered by flies and showed more tail‐swishing than sympatric species (Caro, 2016).

Recent papers have focussed mainly on tabanids and also on *Stomoxys* flies. White horses *Equus caballus* have been found to be less attractive to tabanids than brown horses (Horváth *et al*., 2010). Spotted cow *Bos taurus* models with varying sizes of brown spots on a white background attracted fewer tabanids than uniform brown models, with the numbers of tabanids decreasing with the size of the spots (Blaho *et al*., 2012). Three recent articles have assessed tabanid approach flights and landings using detailed video with captive plains zebras, different coloured horses and horses wearing coats of various patterns (Caro *et al*., 2019, 2023; How *et al*., 2020). These experiments were performed in Europe where tabanids are likely adapted to land on surfaces visually similar to their normal non‐striped hosts. Similar numbers of tabanids approached from a distance but significantly fewer tabanids landed on zebras and horses wearing striped coats. However, contrasting checkered patterns had an equal effect (Caro *et al*., 2019, 2023; How *et al*., 2020). Recently, for *Stomoxys* flies in Kenya, Tombak *et al*. (2022) found a significant preference for landing on impala pelts compared to zebra pelts, and if flies did land on the zebra pelt, they were more likely to land on a black stripe.

### (4) Fly landing deterrence mechanism

Various mechanisms have been suggested for fly deterrence by stripes. These include the aperture effect and aliasing, both of which are optical illusions that could interfere with a fly's visual assessment on final approach to a host. The aperture effect is the ‘barber pole’ illusion whereby the stripes create an illusion of movement in a different direction. This has now been discounted by How *et al*. (2020). Aliasing, whereby parallel stripes are falsely superimposed to create misjudgement of distance, has been discounted by Caro *et al*. (2023). Crypsis is an alternative suggested mechanism if the stripes break up the shape of the host or interfere with the host's polarising light signature. Polarising light interference had also been excluded by Caro *et al*. (2023) and Britten, Thatcher & Caro (2016) but this exclusion was discounted by Horváth (2024) as pelage had been assessed from only one viewing angle, whereas a fly would visualise from multiple viewing angles on approach to a host. So, a form of crypsis (perhaps also involving polarised light) remains the favoured mechanism.

### (5) Blood‐vessel‐masking sub‐hypothesis

Most recently, two articles have suggested that differential heating at the edge of contrasting stripes could mask the heat signature from underlying blood vessels when tabanids are seeking a blood meal (Takács *et al*., 2022; Száz *et al*., 2023). However, tabanids are known to cut the skin with their laciniae (knife‐like mouthparts) (Mullens, 2018) and feed on the pool of blood (a technique known as telmophagy), as opposed to targeting specific vessels (solenophagy), as seen in mosquitos (Diptera: Culicidae) (Baldacchino *et al*., 2014). Pool‐feeding utilises capillaries, which are small at 10 μm diameter and numerous (e.g. in human skin there are 1500–6500 per square cm) (Deegan & Wang, 2019) meaning individual vessels are not specifically targeted. Glossinids and *Stomoxys* are also pool feeders (Krenn & Aspöck, 2012; Lahondère & Lazzari, 2015). By contrast, solenophagous mosquitos target slightly larger vessels of 40–50 μm diameter (approximately twice the size of their piercing fascicle) of which there are 130 vessels per square cm in human skin (Choumet *et al*., 2012; Nixon, 2016).

As tabanids, glossinids and *Stomoxys* flies are pool feeders, they do not need to identify specific vessels. However, if this theory was correct and vessels did need to be identified, only a tiny proportion of hide at the edges of stripes would be protected. Much narrower stripes would be required as the blood vessels are very small, however, very narrow stripes might also reduce differential heating (see thermoregulation hypothesis in Section III). Also the research above (Caro *et al*., 2019, 2023; How *et al*., 2020) found that biting flies were less likely to land on striped surfaces rather than landing and being unable to locate a blood vessel. In summary, we find this blood‐vessel‐masking sub‐hypothesis of the fly hypothesis implausible as tabanids are pool feeders and do not need to target individual vessels and for solenophagous fly species the large difference in scale between stripes and very small skin blood vessels is inconsistent.

Table 2 provides comments on the compatibility of the biting fly hypothesis with our list of zebra stripe features from Table 1.

**Table 2:** Biting fly hypothesis compatibility with zebra stripe features.

No.	Stripe feature question	Comment
1	Is a striping pattern, as opposed to another contrasting pattern, compatible?	Possibly, but various other contrasting non‐striped patterns such as spots and checks (particularly finer patterns) were found to have a similar effect for tabanids (Blaho *et al*., 2012; Caro *et al*., 2019, 2023; How *et al*., 2020)
2	Are black and white the most compatible colours?	Yes, black stripes on white backgrounds have been mainly tested as opposed to other colours of stripes. Brown and white spotty patterns were also effective (Blaho *et al*., 2012). All‐white horses/models were also less preferred (Egri *et al*., 2012; Caro *et al*., 2019).
3	Are stripe contrast and sharpness compatible?	Yes, high stripe contrast has been shown to enhance effect (Caro *et al*., 2023). Edge sharpness enhances contrast. Also, it has been suggested that stripe edges could conceal the heat signature of blood vessels due to the differential temperature of black and white stripes (Takács *et al*., 2022).
4	Are black stripe widths for each body area compatible?	Poor fit, tabanids found to be better deterred by narrow stripes (Egri *et al*., 2012), so it is unclear why some stripes are thicker. Stripe widths are highly variable, if stripes interfere with the ability of flies to land, one might expect an optimum width (see Fig. 1). Also, large black areas are known to be attractive. However, for *Stomoxys* flies, Tombak *et al*. (2022) found no difference in deterrence between wide plains zebra stripes and narrow Grevy's zebra stripes.
5	Is the ratio of black stripe width to white stripe width compatible?	Unknown, often tested at 1:1 ratio with equal widths.
6	Is stripe angle compatible?	Unknown, horizontal, vertical or single angle mainly tested alone, no comparison of different angles.
7	Is stripe upper body stripe distribution compatible?	Yes, stripes are widely distributed which could help protect maximum area from biting flies.
8	Is head and neck stripe distribution compatible?	Yes, protect maximum area from biting flies.
9	Is belly striping compatible?	Poor fit, stripes weaken on belly of Burchell's southern subspecies of plains zebra, and absent on belly of mountain zebra and Grevy's zebra, yet biting flies are known to approach and feed at low level with several tabanid species favouring the belly for feeding (Phelps & Holloway, 1990)
10	Is leg striping compatible?	Poor fit, leg striping weak or absent on Burchell's subspecies of plains zebra, particularly on inner aspect. Legs are frequently targeted by biting flies (Phelps & Holloway, 1990; Muzari *et al*., 2010) although not by all species. In the UK, the tabanid *Haematopota pluvialis* was found to land on the legs on only 11.5% of landings but for *Tabanus bromius*, 83% of landings were on legs or belly (Caro *et al*., 2022). In Sudan >80% of tabanid landings were on lower torso and legs (Mohamed‐Ahmed & Mihok, 2009).
11	Is hindquarter striping compatible?	Possibly, stripes are wider than optimal from experiments, but still in a width range where flies deterred (Egri *et al*., 2012; Caro *et al*., 2014).
12	Is the striped mane compatible?	Poor fit, zebra have a large erect striped mane, the mane is not vulnerable to biting flies. It is possible that a striped mane could deter flies from landing nearby.
13	Is the striped tail compatible?	Yes, stump of tail vulnerable to flies.
14	Is zebra behaviour compatible?	Yes, zebra move away from biting flies which is compatible with trying to avoid being bitten.
15	Does the hypothesis explain why only zebra are striped among sympatric African herbivores?	Poor fit, extra vulnerability to diseases and short hair suggested to increase vulnerability to flies (see Section VII.1).
16	Does the hypothesis explain why equids elsewhere are not striped?	Possibly, if under less harassment from biting flies or if less disease carried by flies.
17	Does the hypothesis explain the gradient of increasing stripe vividness from south to north?	Possibly, as biting flies more populous in the north.
18	Does the hypothesis fit for mountain zebra and Grevy's zebra (as well as the plains zebra)?	Poor fit, mountain zebra and Grevy's zebra live in more arid climates where there is less biting fly challenge (Joubert, 1972).
19	Does the hypothesis have supporting data?	Yes, extensive data for various dipterans not preferring or avoiding stripes. But all‐white, spots and checkerboard pattern also less preferred.

## III. THERMOREGULATION

Thermoregulation has been suggested as a possible driver for zebra stripes: the hypothesis being that differential heating of black and white stripes causes eddy air currents that increase cooling by sweat evaporation (Morris, 1990; Cobb & Cobb, 2019). During sunlight, black stripes have been found to be 12–15 °C higher in temperature than white stripes (Cobb & Cobb, 2019) and zebra are water dependent and cool by sweating (Fuller *et al*., 2000; Cain, Owen‐Smith & Macandza, 2012; Veldhuis *et al*., 2019). It has been suggested that equids might be more susceptible to heating as they spend more time foraging due to their hindgut digestive physiology (Cobb & Cobb, 2019). The increased intensity of plains zebra striping in more northern subspecies has been suggested to correlate best with increasing temperature (Larison *et al*., 2015). However, zebra foraging activity has not been found to be influenced by thermal constraints (Owen‐Smith & Goodall, 2014). Horváth *et al*. (2018) used barrels covered with striped hides and found no evidence of cooling but the hides were dry so evaporative cooling could not be explored. Pereszlényi *et al*. (2021) used schlieren imaging and found the effect of stripes on upwelling air streams was negligible, with similar airflow over white or black horse hides and the hypothesised cooling eddies did not form; also any small air movements would be overwhelmed by even light wind or movement of the animal.

Table 3 provides an assessment of the compatibility of the thermoregulation hypothesis with our list of zebra stripe features.

**Table 3:** Thermoregulation hypothesis compatibility with zebra stripe features.

No.	Stripe feature question	Comment
1	Is a striping pattern, as opposed to another contrasting pattern, compatible?	Possibly, but other contrasting patterns could have similar effect.
2	Are black and white the most compatible colours?	Yes, for heating differential.
3	Are stripe contrast and sharpness compatible?	Yes, contrast might cause differential heating which could help if cooling eddies were formed. Stripes would need to be reasonably sharp for differential heating.
4	Are black stripe widths for each body area compatible?	Poor fit, if there is an optimum stripe width for cooling, one might expect more uniform width. Very thin stripes on face seem unlikely to function as suggested. No current evidence for optimum stripe width for this function.
5	Is the ratio of black stripe width to white stripe width compatible?	Poor fit, equal white and black widths might be better. Narrow white stripes on neck seem unlikely to function as suggested.
6	Is stripe angle compatible?	Unknown.
7	Is stripe upper body stripe distribution compatible?	Possibly, widespread stripes on upper body would be consistent with hypothesis, as exposed to the sun, but dorsal stripes have a greater proportion of black which would encourage heating.
8	Is head and neck stripe distribution compatible?	Possibly, widespread stripes would be consistent with hypothesis as exposed to the sun. However, stripes on underside of head and neck are a poor fit.
9	Is belly striping compatible?	No, stripes on belly do not support hypothesis, as not in the sun during hottest parts of the day when the sun is overhead. Zebra infrequently lie down (when the belly would be partly in sun) (Joubert, 1972; Regassa & Solomon, 2014; Forbes & Kerley, 2022).
10	Is leg striping compatible?	Yes, stripes on legs consistent if in the sun. Inner aspect of legs not striped – consistent, as not in sun.
11	Is hindquarter striping compatible?	Unknown, stripes wider, might expect optimum width.
12	Is the striped mane compatible?	Poor fit, mane hair does not require cooling. Possibly could enhance sweat evaporation.
13	Is the striped tail compatible?	No, constant swishing would disrupt any differential air currents on the tail itself and on the hindquarters.
14	Is zebra behaviour compatible?	Unknown.
15	Does the hypothesis explain why only zebra are striped among sympatric African herbivores?	Poor fit, if effective why are no other species in hot climates striped? Equids could be more susceptible to heating due to hind‐gut physiology or for another reason.
16	Does the hypothesis explain why equids elsewhere are not striped?	Yes, if living in less hot environments. However, the African wild ass (*Equus africanus*) lives in hot desert and is not striped.
17	Does the hypothesis explain the gradient of increasing stripe vividness from south to north?	Possibly, due to temperature, but more northern subspecies also have belly striping despite not being exposed to the sun.
18	Does the hypothesis fit for mountain zebra and Grevy's zebra (as well as the plains zebra)?	Poor fit, mountain zebra live in a cooler climate and have strong striping. The Grevy's zebra lives in a hotter climate, its stripes are much narrower which weakens this hypothesis as one would predict an ideal stripe width.
19	Does the hypothesis have supporting data?	No.

## IV. CRYPSIS

An original hypothesis suggested the stripes could merge with long grass to provide camouflage (Thayer, 1909), but this subsequently has been contradicted by multiple authors. Zebras do not display behaviour associated with crypsis; they do not attempt to hide and do not freeze motionless when predators are present (Ruxton, 2002; Ioannou & Krause, 2009). The bold diagonal contrasting pattern is eye‐catching and conspicuous at short distance (see Section VII.5) which is not consistent with crypsis at this distance. Also many zebra have a continuous black line on the edge of mane (Fig. 1), which would prevent stripes acting as disruptive camouflage (Stevens *et al*., 2006; Troscianko, Skelhorn & Stevens, 2017). Melin *et al*. (2016) assessed the distance from which zebra stripes could be resolved by humans in various lights and then extrapolated their results for lion (*Panthera leo*) vision; they found the stripes could not be resolved at distance (maximum distance for a lion to visualise plains zebra stripes was estimated at 80/46/11 m for daytime/dusk/night) and therefore they concluded the stripes did not evolve primarily for crypsis. This research was interesting, but their conclusion seems open to question – concluding the stripes do not function for crypsis (i.e. not to be seen) because they could not be seen. This logic could apply if the stripes acted as disruptive camouflage but not for background matching, countershading for self‐shadow concealment (Stevens & Merilaita, 2009) or if the stripes are not resolved at distance and therefore become an average grey colour. Grey colouration may possibly be good for crypsis, however, this could also be achieved without stripes by being a similar uniform grey colour.

A crypsis function potentially would be significantly enhanced in low light and at night due to reduced visual input for the observer. However, the bright white stripes might remain conspicuous at short distance.

In Grevy's zebra and to a lesser extent in mountain zebra, the black stripes are narrower and there is also a higher black to white stripe ratio and both of these stripe attributes reduce relative conspicuousness, meaning they will average to grey at a relatively shorter distance than for plains zebra, and the shade of grey will be darker. These features will make Grevy's and mountain zebra possibly cryptic at relatively shorter distances. Both of these species also have white bellies, which could be consistent with crypsis through countershading (Rowland *et al*., 2007; Stevens & Merilaita, 2009).

Table 4 provides an assessment on the compatibility of the crypsis hypothesis with our list of zebra stripe features.

**Table 4:** Crypsis hypothesis compatibility with zebra stripe features.

No.	Stripe feature question	Comment
1	Is a striping pattern, as opposed to another contrasting pattern, compatible?	Possibly at distance, when the striping cannot be resolved and the visualised colour becomes an average grey. Striping good for averaging at distance beyond the grid resolution of the observer, whereas, for example, large spots would not average evenly.
2	Are black and white the most compatible colours?	Poor fit, less‐contrasting colours would be less conspicuous at closer distances. Brown tinge in young foals possibly more cryptic.
3	Are stripe contrast and sharpness compatible?	Poor fit, contrast and sharpness make stripes more conspicuous. Less sharp stripes in young foals possibly more cryptic.
4	Are black stripe widths for each body area compatible?	Poor fit, not clear if optimum width for crypsis, wider stripes visible from further away. Narrower stripes could be cryptic from distance due to colour averaging.
5	Is the ratio of black stripe width to white stripe width compatible?	Poor fit, plains zebra ratio with wide black stripes and equal or wider white stripes particularly on the hindquarters, favours conspicuousness (Iizuka *et al*., 2021). However, wider black stripe with relatively narrower white stripe would reduce conspicuousness and make the animal relatively more cryptic (as seen in the neck and flanks of Grevy's and mountain zebra and even more so on the neck of the extinct *quagga* subspecies of plains zebra *Equus quagga quagga*). This ratio will also dictate the shade of grey at distance.
6	Is stripe angle compatible?	Unknown.
7	Is stripe upper body stripe distribution compatible?	Yes, widespread stripes on upper body and head would be consistent with hypothesis if effective as crypsis.
8	Is head and neck stripe distribution compatible?	Yes, black to white ratio higher on neck which reduces conspicuousness relative to rear flank.
9	Is belly striping compatible?	Yes, black stripes taper towards the belly which could help provide countershading as would the absence of belly stripes in Burchell's southern plains zebra subspecies.
10	Is leg striping compatible?	Yes, widespread stripes consistent if effective as crypsis. Lack of stripes on inner legs also consistent as not visible. As observer moves to distance, the narrow stripes on leg and head become more difficult to resolve (see Fig. 2).
11	Is hindquarter striping compatible?	Poor fit, wide stripes conspicuous from greater distance (Muhl‐Richardson *et al*., 2021).
12	Is the striped mane compatible?	No, enlarges silhouette and so not cryptic and increases conspicuousness.
13	Is the striped tail compatible?	No, constant movement compromises crypsis. Black tail is conspicuous against large hindquarter white stripes.
14	Is zebra behaviour compatible?	No, zebra do not freeze or hide, and use an array of vocalisations (including alarm calls in response to nearby predators).
15	Does the hypothesis explain why only zebra are striped among sympatric African herbivores?	No.
16	Does the hypothesis explain why equids elsewhere are not striped?	Possibly, if less predation pressure from ambush predators.
17	Does the hypothesis explain the gradient of increasing stripe vividness from south to north?	No.
18	Does the hypothesis fit for mountain zebra and Grevy's zebra (as well as the plains zebra)?	Yes, narrower stripes and higher black to white stripe ratio in Grevy's zebra and mountain zebra and these stripe attributes reduce relative conspicuousness, meaning the stripes will average to grey at relatively shorter distance.
19	Does the hypothesis have supporting data?	No.

## V. DAZZLE COLOURATION

The brightly contrasting zebra stripes have been hypothesised to cause a visual confusion effect for a predator selecting an individual prey animal. How & Zanker (2014) suggested this could be *via* an effect similar to the visual illusions of the ‘wagon‐wheel’ spatiotemporal aliasing effect or the ‘barber‐pole’ illusion/aperture effect. These illusions are exaggerated in video sequences by the lack of three‐dimensional cues and the stroboscopic nature of video capture (How & Zanker, 2014). In simulated predator chases using human subjects, accurate perception of trajectory and speed have been found to be hampered by stripes both parallel and perpendicular to the direction of movement (Hogan, Cuthill & Scott‐Samuel, 2016; Kodandaramaiah *et al*., 2020). Most zebra stripes are perpendicular to the overall direction of movement, but movement of most zebra body parts is non‐linear due to the complex gaits of equine locomotion. Some investigators found lower contrast patterns harder to capture (Stevens *et al*., 2011) and others high‐contrast patterns (Scott‐Samuel *et al*., 2011; Kodandaramaiah *et al*., 2020). Some found stripes effective (Kodandaramaiah *et al*., 2020) and some zigzags or checked patterns (Scott‐Samuel *et al*., 2011). Overall results are conflicting (Scott‐Samuel *et al*., 2023). There have been relatively few possible examples of dazzle colouration published. Recent papers have focussed on longitudinal stripes: in lizards to deflect attention to the tail (which may break off) (Murali, Merilaita & Kodandaramaiah, 2018) and in snakes to mask body speed and change in direction (Brodie, 1992). This effect appears particularly effective for snakes where a longitudinal line combined with the lack of legs or perpendicular speed reference marks and the continuous concealed nature of snake locomotion provide no cues for a predator to judge body speed. However, this effect cannot occur for zebra due to stripes perpendicular to motion and anatomical movement cues.

The suggested mechanism might possibly be more effective in low light or night due to reduced total visual input for a predator, but zebra predators do have excellent night vision.

Table 5 provides an assessment of the compatibility of the dazzle colouration hypothesis with our list of zebra stripe features.

**Table 5:** Dazzle colouration hypothesis compatibility with zebra stripe features.

No.	Stripe feature question	Comment
1	Is a striping pattern, as opposed to another contrasting pattern, compatible?	Possibly, poorly understood process with limited examples.
2	Are black and white the most compatible colours?	Possibly, if high‐contrast pattern helpful.
3	Are stripe contrast and sharpness compatible?	Possibly, however some experiments found targets with low‐contrast patterns were harder to capture (Stevens *et al*., 2011).
4	Are black stripe widths for each body area compatible?	Unknown, not clear if there is an optimum width for effect.
5	Is the ratio of black stripe width to white stripe width compatible?	Unknown.
6	Is stripe angle compatible?	Possibly, horizontal stripe better for masking trajectory and speed (Hogan *et al*., 2016), rear stripes almost horizontal. But neck and other striping perpendicular to movement.
7	Is stripe upper body stripe distribution compatible?	Yes, widespread stripes on upper body would be consistent with hypothesis if effective as dazzle colouration.
8	Is head and neck stripe distribution compatible?	Poor fit, as suggested function applies more for a confusion effect for fleeing zebra, where the hindquarters, body and legs would be more relevant.
9	Is belly striping compatible?	Unknown.
10	Is leg striping compatible?	Yes, lack of stripes on inner aspect consistent.
11	Is hindquarter striping compatible?	Yes.
12	Is the striped mane compatible?	Unknown.
13	Is the striped tail compatible?	Unknown. Tail constantly swishing.
14	Is zebra behaviour compatible?	Possibly, as zebra stay close together in small herds which might enhance confusion effect. However, they often flee separately (Caro, 2016).
15	Does the hypothesis explain why only zebra are striped among sympatric African herbivores?	No.
16	Does the hypothesis explain why equids elsewhere are not striped?	Possibly, if under less predation pressure.
17	Does the hypothesis explain the gradient of increasing stripe vividness from south to north?	No.
18	Does the hypothesis fit for mountain zebra and Grevy's zebra (as well as the plains zebra)?	Possibly, if function effective.
19	Does the hypothesis have supporting data?	No.

## VI. INTERSPECIFIC SIGNALLING FOR MIXED‐SPECIES HERDING

The stripes have been hypothesised to act as a form of interspecies attraction signal to facilitate mixed‐species herding (Ireland & Ruxton, 2017). Mixed‐species herding can reduce the risk of predation, mainly from lions, and lions account for a large proportion of plains zebra mortality (Sinclair, 1985; Grange & Duncan, 2006; Grange *et al*., 2015). Plains zebra are more likely to be found in mixed‐species herds when the risk of predation is higher (Stears *et al*., 2020; Kiffner *et al*., 2022). Lions are ambush predators and rely on surprise and rapid acceleration, whereas zebra are faster after the initial acceleration phase (Elliott, Cowan & Holling, 1977). In an open landscape, a zebra is safest in the centre of a herd where a lion cannot stalk close enough to utilise surprise. Plains zebra have stable core social groups known as harems consisting of one stallion, several females and their young (Estes, 1991; Fischhoff *et al*., 2007a; Grange *et al*., 2015). Zebra can reduce predation risk by herding in larger groups when predation risk is high or dispersing as a harem when risk is lower.

Mixed‐species herds can reduce predation risk by increasing group size known as the encounter‐dilution effect (Turner & Pitcher, 1986) and the ‘many‐eyes’ effect of shared vigilance (Beauchamp, 2014). However, there are nutritional costs in avoiding predation (Barnier *et al*., 2014) as increasing group size increases feeding competition and reduces freedom in patch and grass choice. Mixed‐species herds can mitigate feeding competition as different species target different plants or plant parts. This is particularly the case for mixed‐species herds containing zebra, as zebra are hind‐gut fermenters and seek different forage from bovid ruminants (all zebra co‐herding ungulates are ruminants). Zebra prefer combinations of longer browner grass and short green grass, whereas ruminants such as blue wildebeest prefer shorter greener grass (Sinclair, 1985; Mandlate, Arsenault & Rodrigues, 2019). With their opposing incisors (ruminants only have lower incisors), zebra can also clip tall grass stems and eat grass seeds (Owaga, 1975; McNaughton, 1985; Jong & Prins, 2023). This is a form of resource partitioning and has been observed to happen on a small spatial scale as ungulates feed together, particularly during the wet season (Owaga, 1975).

For zebra, mixed‐species herds have multiple further advantages in comparison to single‐species herds including increased feeding efficiency due to reduced vigilance (Schmitt, Stears & Shrader, 2016; Stears *et al*., 2020), increased predator detection due to differing heterospecific sensory skills and associated heterospecific alarm calls (Palmer & Gross, 2018; Meise, Franks & Bro‐Jørgensen, 2020), lower parasite levels (Odadi *et al*., 2011) and less competition for zebra mates (Stears *et al*., 2020).

For sympatric ruminants, mixed‐species herding can also reduce predation risk (Stears *et al*., 2020) and mitigate nutritional costs through improved feeding efficiency (due to reduced vigilance) and differing diets. Further, some ruminants may prefer to herd with zebra as zebra provide additional benefits: there will be a greater reduction in feeding competition due to zebra hindgut digestion and increased feeding efficiency due to zebra vigilance. Zebra likely have better visual acuity than associating ruminants (Veilleux & Kirk, 2014; Melin *et al*., 2016) and adult zebras take turns being vigilant. Zebra also spend less time resting or lying down than ruminants – zebra only rest in the heat around noon when lions are less active (Klingel, 1967; Neuhaus & Ruckstuhl, 2002; Regassa & Solomon, 2014). Wildebeest and impala have been shown to eavesdrop on zebra alarm calls and they considered zebra alarm calls the most reliable alarm call (Palmer & Gross, 2018). Further benefits are the aggression of the zebra stallion to some predators such as African wild dogs *Lycaon pictus* and single hyenas *Crocuta crocuta* (Estes, 1991) and the experience of the zebra mare in leading – each harem is led by the most experienced mare and the stallion protects the rear (Estes, 1991).

As both zebra and associating ruminants can gain from herding together it is a mutualistic relationship. Plains zebra have been found to associate with multiple sympatric ruminant herbivores but particularly blue wildebeest, eland *Taurotragus oryx*, Cape buffalo, Grant's gazelle *Nanger granti*, Thomson's gazelle and giraffe (Kiffner *et al*., 2014; Schmitt *et al*., 2014). They form the strongest association with wildebeest (Anderson *et al*., 2016; Meise *et al*., 2020; Kiffner *et al*., 2022). Zebra are one of the species found most often in mixed‐species herds (Beaudrot *et al*., 2020). The food competition costs of mixed‐species herding are relatively lower in the wet season meaning species have stronger associations and are more commonly found in mixed‐species (and single‐species) herds in the wet season (Kiffner *et al*., 2014).

During the fusion of zebra and ruminant mixed‐species herds in response to a perceived increase in predation risk, conspicuous stripes could help ruminants to locate and identify zebra at short to medium distance (at less than about 100 m in daytime). As well as being conspicuous, the stripes fulfil the criteria for a highly effective signal. High‐contrast repeating patterns such as stripes are common in visual signalling (Kenward *et al*., 2004; Stevens & Ruxton, 2012). The principal features of a visual signal are detectability (visibility, conspicuousness and contrast), memorability, discriminability and the systems concepts: redundancy, degeneracy and pluripotentiality (Guilford & Dawkins, 1991; Wiley, 2006; Chen & Crilly, 2014; Hebets *et al*., 2016). Zebra pattern is visible from all directions, contrasts with light and dark backgrounds and provides high internal contrast (Wertheim, 2010). In low light, the white stripes will reflect light and enhance visibility (Ortolani, 1999). Diagonal stripes are the most conspicuous (to humans) (Iizuka *et al*., 2021). The erect striped mane enlarges the signal. Stripes perpendicular to movement enhance conspicuousness and the repeating pattern provides memorability (Guilford & Dawkins, 1991). The zebra has excellent discriminability being clearly recognisable and different from all sympatric species. For sympatric African ungulates, which are all dichromats (Jacobs, 1993), the zebra is likely the most conspicuous mammal for its size at short to medium distance. Colour would only enhance the signal for tri‐ or tetrachromats. Mobile non‐hiding behaviour and tail‐swishing also improve conspicuousness. The all‐over pattern provides redundancy if only a fragment is visible; zebra shape and mane provide degeneracy to allow identification in silhouette when stripes are not visible and noisy vocalisations can also enhance discoverability and discriminability when the entire animal is not visible (zebra have been found to be significantly more noisy than impala or topi *Damaliscus lunatus jimela*; Caro, 2016). The potential for multiple signal receivers provides pluripotentiality.

Table 6 provides an assessment of the compatibility of the interspecific signalling for mixed‐species herding hypothesis with our list of stripe features.

**Table 6:** Interspecific signalling for mixed‐species herding hypothesis compatibility with stripe features.

No.	Stripe feature question	Comment
1	Is a striping pattern, as opposed to another contrasting pattern, compatible?	Yes, repeating stripe patterns are common for signalling (Stevens & Ruxton, 2012) and enhance memorability. Stripes have coherent edges which are prioritised or enhanced by mammalian visual processing.
2	Are black and white the most compatible colours?	Yes, as maximise contrast with background and maximise internal contrast.
3	Are stripe contrast and sharpness compatible?	Yes, high contrast and sharp stripes increase conspicuousness.
4	Are black stripe widths for each body area compatible?	Possibly, thicker stripes on mobile hindquarters can increase conspicuousness and allow following from a greater distance. Narrow stripes on face could be helpful (at close distance) so that multiple stripes remain visible to retain consistent repeating pattern.
5	Is the ratio of black stripe width to white stripe width compatible?	Possibly, wider or equal white stripes than black on hindquarters which should increase conspicuousness (Iizuka *et al*., 2021) and may assist following, particularly in low light (Guthrie, 1971). Also, wider white stripes relative to black stripes on head make head conspicuous in low light. On neck, black stripes are wider than white and so visible at shorter distances.
6	Is stripe angle compatible?	Yes, diagonal stripe most conspicuous (for humans) (Iizuka *et al*., 2021). Striping perpendicular to direction of movement will also increase conspicuousness.
7	Is stripe upper body stripe distribution compatible?	Yes, stripes visible from all directions and redundancy if part of zebra obscured.
8	Is head and neck stripe distribution compatible?	Yes, stripes visible from all directions. Neck is high and wide and good for species recognition even in tall vegetation. Neck provides conspicuous diagonal striping: diagonal in one direction with head elevated and diagonal in the other direction when grazing.
9	Is belly striping compatible?	Possibly, increases size and effectiveness of signal and redundancy. Least visible body part for signalling and so might expect weaker striping, however, as the belly is curved, lowermost tip of belly is still visible from side.
10	Is leg striping compatible?	Yes, increase size and effectiveness of signal and redundancy. Lack of stripes on inner aspect consistent.
11	Is hindquarter striping compatible?	Yes, hindquarters most conspicuous part and from the greatest distance (Muhl‐Richardson *et al*., 2021). Possibly useful for following or leadership role. Plains, mountain and Grevy's zebra all have different hindquarter patterns around base of tail which would also allow conspecific identification and following in large mixed‐species zebra herds (although currently limited sympatry between zebra species).
12	Is the striped mane compatible?	Yes, increases size of signal. Mane is large and erect but erect mane also present in other non‐striped equids.
13	Is the striped tail compatible?	Yes, frequent or constant tail swishing increases conspicuousness. Black tail tuft conspicuous when moving across white hindquarter stripes.
14	Is zebra behaviour compatible?	Yes, zebra are non‐aggressive to other species. Zebra remain conspicuous and do not hide. Zebra remain in small groups meaning other species are joining a small herd. Zebra are found in mixed‐species herds in times and places of increased predation risk (in wet season when abundant food and less competition, zebra are also found in large single‐species herds).
15	Does the hypothesis explain why only zebra are striped among sympatric African herbivores?	Yes, sympatric African similarly sized bovid herbivores are ruminants and would have increased feeding competition if joined by other ruminants. Zebra are less vulnerable to feeding competition due to hindgut digestion and able to target forage unsuitable for ruminants and so the overall benefit of mixed‐species herding is greater.
16	Does the hypothesis explain why equids elsewhere are not striped?	Yes, equids elsewhere unlikely to have sufficient potential sympatric co‐herding species in near vicinity to utilise multi‐species herding and might not be under the same predation pressure.
17	Does the hypothesis explain the gradient of increasing stripe vividness from south to north?	Possibly, currently the total population and number of species of sympatric herbivores are greatest in the north of zebra range with peak in Tanzania/Kenya which maximises the opportunity for multi‐species herding (Turpie & Crowe, 1994; Said *et al*., 2003). Therefore, the opportunities for mixed‐species herding will gradually decrease further south.
18	Does the hypothesis fit for mountain zebra and Grevy's zebra (as well as the plains zebra)?	Possibly, Grevy's and mountain zebra form, and historically formed, mixed‐species groups with other sympatric herbivores and plains zebra in the wet season (Keast, 1965; Estes, 1999) and would at those times benefit from reduced predation risk.
19	Does the hypothesis have supporting data?	No, only indirect data for mixed‐species associations.

## VII. DISCUSSION

The assessment of stripe features highlights areas of poor compatibility with various hypotheses. The ‘no’ answers are most helpful in clarifying the debate as they can point to a potential flaw or limitation in the hypothesis. We will discuss each hypothesis in turn. See Table 7 for a summary comparison of the different hypotheses.

**Table 7:** Comparison of zebra stripe features among hypotheses. Key: yes, compatible; (yes), possibly compatible; —, unknown; (no), poor fit; no, not compatible.

Stripe feature	Biting flies	Thermoregulation	Crypsis	Dazzle colouration	Interspecific signalling for mixed‐species herding
Striped pattern	(yes)	(yes)	(yes)	(yes)	yes
Black and white stripes	yes	yes	(no)	(yes)	yes
Stripe contrast and sharpness	yes	yes	(no)	(yes)	yes
Stripe width	(no)	(no)	(no)	—	(yes)
Stripe ratio	—	(no)	(no)	—	(yes)
Stripe angle	—	—	—	(yes)	yes
Upper body	yes	(yes)	yes	yes	yes
Head/neck	yes	(yes)	yes	(no)	yes
Belly	(no)	no	yes	—	(yes)
Legs	(no)	yes	yes	yes	yes
Hindquarter	(yes)	—	(no)	yes	yes
Mane	(no)	(no)	no	—	yes
tail	yes	no	no	—	yes
Behaviour	yes	—	no	(yes)	yes
Only zebra among sympatric	(no)	(no)	no	no	yes
Only zebra among equids	(yes)	yes	(yes)	(yes)	yes
Stripe gradient	(yes)	(yes)	no	no	(yes)
Mountain and Grevy's zebra	(no)	(no)	yes	(yes)	(yes)
Supporting data	yes	no	no	no	no

### (1) Biting flies

For tabanids and also *Stomoxys* flies, there is extensive evidence (mainly from Europe) demonstrating that stripes (and other contrasting patterns) are a significant deterrent to landing. However, it does not automatically follow that biting fly avoidance is the sole (or even the main) driver for the selection of zebra stripes. For this hypothesis, there are several inconsistencies with stripe features. Prominent striping of the large and erect striped mane is hard to reconcile with the hypothesis as the mane is not vulnerable to flies. Experiments with horses wearing striped coats did not deter landings on the uncovered neck and head, consistent with stripes only influencing the final alighting point (Caro *et al*., 2019). The striped mane could fit if stripes had a signalling function to flies, for example acting as an aposematic signal. However, this has not been considered in the literature as a possibility and seems unlikely (see Section VII.1). The absence of belly and leg striping on some species/subspecies when these areas of the body (for all large mammals, including zebra) are frequently targeted by biting flies is also problematic (e.g. in the UK, for the tabanid *Tabanus bromius*, 83% of landings were on the legs or belly; Caro *et al*., 2022). A further significant challenge for this hypothesis is the lack of another mammal (or any other terrestrial vertebrate) worldwide with striping for a similar purpose when many mammals are bothered by flies and many have other recurrent adaptations to protect themselves such as mobile tails. This issue is emphasised by the multiple other sympatric ungulates living with the same flies without this adaptation. Further, the mountain zebra and Grevy's zebra live in more arid or mountainous areas where there are fewer biting flies (Joubert, 1972).

Regarding this question of why only zebras are strongly striped among large African ungulates, Caro *et al*. (2019) highlight fly‐transmitted diseases and also state that zebra may be more vulnerable due to their short hair (Caro *et al*., 2019). We will discuss these in turn. The effect of fly‐transmitted diseases can be exaggerated by confusing wild zebra that have natural adapted immunity with the high vulnerability of introduced horses that have no natural immunity or physiological defence.

Zebra have evolved with a diversity of microorganisms or microparasites, some of which are transmitted by biting flies. Some of these microorganisms are adapted to utilise zebra as reservoir or ‘natural’ hosts in enzootic cycles (Radcliffe & Osofsky, 2002; Cossu *et al*., 2022). The majority are viruses, but some are bacteria or protozoa. Co‐evolution with these microorganisms can lead to reduced pathogenicity or costs (Favaretto *et al*., 2025), as the microparasites will have a survival benefit if their co‐evolved zebra hosts also survive, are common and have a prolonged mild viraemia to allow ongoing transmission. With some microorganisms, this can lead to a mutualistic relationship (Favaretto *et al*., 2025). A new or naïve host that is related (such as a horse) and therefore susceptible to the same microorganisms can suffer high pathogenicity due to a lack of adapted immunity including mechanisms to regulate virus replication and can act as an accidental, incidental or dead‐end host (as the animal does not survive to allow ongoing microorganism transmission) (Weaver & Reisen, 2010; Sack *et al*., 2020).

Caro *et al*. (2019, p. 2) state that tabanids carry diseases ‘fatal to zebras including trypanosomiasis, equine infectious anaemia, African horse sickness, and equine influenza’. For trypanosomiasis, equine infectious anaemia and African horse sickness, zebra can act as protozoan/viral reservoirs with low pathogenicity to the host zebra but can be a source of serious infection for domestic animals that are more vulnerable. Trypanosomiasis (protozoan) causes mild or no disease in zebra (Mulla & Rickman, 1988a,b). For equine infectious anaemia (viral), equids are the only host, and most horses and zebra show no sign of the disease (Foil, 1989; Cook *et al*., 2001). African horse sickness (viral) affects domestic equids, zebra are resistant and rarely show signs of infection (occasionally a mild fever) and generally act as asymptomatic hosts; the main vectors are *Culicoides* biting midges rather than tabanids (Radcliffe & Osofsky, 2002; Mellor & Hamblin, 2004; Carpenter *et al*., 2017; Dennis *et al*., 2019). Equine influenza (influenza A virus) has a dual host tropism with a background reservoir in waterfowl (van Maanen & Cullinane, 2002). It mainly affects racehorses and show‐horses which are transported frequently and are kept in close proximity (van Maanen & Cullinane, 2002) and is not a significant issue for wild zebra (Barnard, 1997; Guthrie, Stevens & Bosman, 1999). Also it is a more modern disease increased by human activities (Gibbs & Anderson, 2010) and therefore is unlikely to have contributed to the evolution of stripes.

Caro *et al*. (2022) provide a longer list of diseases ‘transmitted to ungulates’ by tabanids, glossinids and *Stomoxys* flies, of which they state that equine infectious anaemia, vesicular stomatitis, trypanosomiasis, anthrax, tularaemia, anaplasmosis, African horse sickness, encephalitis and West Nile fever are ‘dangerous or lethal to equids’ (Caro *et al*., 2022, p. 28). The inclusive term ‘equid’ is potentially misleading as these are mainly diseases dangerous or lethal to domestic horses (or other mammals), and not zebra. Trypanosomiasis, equine infectious anaemia and African horse sickness were discussed above. Vesicular stomatitis (viral) affects cattle and horses and is geographically restricted to North and South America (Hubálek, Rudolf & Nowotny, 2014). West Nile Fever (viral) is an emerging infectious disease and has been dated to an origin in the 16th or 17th century; it is mainly carried by mosquitos and the main hosts are wild birds but it can affect horses as an accidental or dead‐end host (Tompkins *et al*., 2015; Mencattelli *et al*., 2022). Tularemia (bacterial) is spread by mosquitos, tabanids and ticks (Ixodidae); it affects many wild and domesticated animals including horses and occurs in North America, Scandinavia and north Asia, but not in Africa (Sjostedt, 2007). Anaplasmosis (bacterial) is mainly spread by ticks and causes a self‐limiting infection in horses and zebra. It is most prevalent in Europe and North America, but plains zebra have been found to have antibodies (Ngeranwa *et al*., 2008; Saleem *et al*., 2018). Encephalitis (equine) (viral) affects horses in North and South America (Hubálek *et al*., 2014). Anthrax (bacterial) is spread by spores but also by flies such as tabanids and currently causes regular outbreaks of zebra mortality. However, it is thought to have evolved in the mid‐Holocene and its incidence has been increased by man (Van Ert *et al*., 2007). The opportunities for many of these diseases have been increased by human activities by introducing, moving, overcrowding and manipulating domestic animals (inbreeding and low genetic diversity) and creating unnatural species proximity (Tompkins *et al*., 2015).

Thus, by equating wild zebra with domestic horses, or using inclusive terms like ‘equids’, zebra are implied to be vulnerable to many serious infectious diseases transmitted by biting flies. However, most of these diseases are potentially serious for horses rather than zebra, or are not prevalent in Africa. Furthermore, some diseases are emergent or have been increased by man (Williams *et al*., 2002; Petersen, Petrosillo & Koopmans, 2016). Overall, many of the diseases potentially carried by biting flies cannot be used to explain zebra stripes because they either do not cause significant zebra morbidity or mortality, are not present in Africa, or post‐date stripe evolution. During the evolution and era of striped zebra, zebra will have formed an immunological balance and genetic equilibrium (Hurst, 2021) with various sympatric infectious microorganisms; this will have some costs even if zebra are resistant or tolerant of infection, but it is difficult to use these diseases to justify why zebra are the only striped ungulate. There is no evidence to suggest that zebra are more vulnerable (and they are unlikely to be more vulnerable) to fly‐carried diseases than sympatric ungulates. For viruses, a comparative review of antigens/antibodies detected in African ungulates found buffalo had been exposed to the most viruses, followed by blue wildebeest, impala and warthog (Swanepoel, Crafford & Quan, 2021).

The other reason that has been given for particular zebra vulnerability to biting flies is short hair; zebra have shorter hair than sympatric mammals (Caro *et al*., 2014). The short hair argument is also not compelling: if flies were important to avoid, longer hair could be adapted in a relatively short space of time. Waterbuck *Kobus ellipsiprymnus*, which live close to water where there are higher numbers of biting flies, have skin odours that repel flies and have longer hair (Gikonyo *et al*., 2002; Caro *et al*., 2014). Zebra also have odours found to be less attractive to glossinids (Olaide *et al*., 2019) (zebra are non‐preferred hosts) but have short hair (Caro *et al*., 2014). If zebra pelage is adapted to protect from flies and if short hair increases vulnerability to flies, having short hair which increases vulnerability and stripes to reduce vulnerability seems unlikely, unless there is a net greater advantage for zebra to have shorter hair than sympatric mammals. If, for another reason, perhaps for thermoregulation, zebra were under adaption pressure to have short hair, then that adaption pressure would have to be greater than the adaption pressure to deter flies (if short hair increases vulnerability to flies). A more straightforward reason for the short hair is to maintain the sharpness of the stripes, as long hair would blur the edges, and the stripes have a purpose other than, or additional to, deterring flies. Therefore, the stripes may require the short hair, rather than the short hair requiring the stripes.

A match between zebra range and the predicted overlapping range of tabanids and glossinids is another reason given to support the hypothesis. However, this might be expected as tabanids and glossinids require hosts and so the flies could be matching total host abundance. Glossinids are entirely haematophagous and can only occur where there are available hosts; tabanids also feed from flowers which will likely match with available vegetation for herbivores, and also tabanids and glossinids require access to water which can also match with suitable habitat for herbivores. Invertebrate species richness has also been found to follow total species richness (Hawkins *et al*., 2003).

There also has been little consensus regarding the underlying reason why a fly does not land on a striped surface. Does the fly not recognise a striped target as a host? If so, the stripes are a form of crypsis, as suggested by Caro *et al*. (2023). This is possible, although African tabanid species or other dipterans that specifically target zebra as hosts are more likely adapted to recognise zebra. Or are biting flies adapted not to land on striped surfaces, e.g. for their own crypsis or because the stripes are an aposematic warning signal? Flies have been shown to prefer landing on black stripes, which could aid their own crypsis or warming for escape, and might avoid white or striped surfaces. An aposematic signal seems unlikely as for such as a signal to have evolutionary stability, and in the absence of another defended species to mimic, it would require the signal to deter flies effectively and it should be to the flies' advantage to follow the warning. No zebra additional defence (e.g. chemical defence) has been suggested or found, zebra are still bothered by flies and it appears to be to the flies' advantage to ignore the signal. Or are stripes less preferred than attractive dark colours as the striped pelage is 50% white? Or finally, do biting flies struggle to land because the stripes disturb the optical processes of landing, perhaps similar to dazzle colouration? However, this does seem relatively implausible as tabanids are known to be agile fliers: females can land on narrow stems and grass blades to lay eggs; males can both drink from pools of water and initiate mating whilst in flight, and both sexes land on small flower heads (Mullens, 2018). In tabanids, the optical aperture effect and spatiotemporal aliasing have been excluded by How *et al*. (2020) and Caro *et al*. (2023). Furthermore, research to assess these factors would ideally take place in Africa with fly species sympatric with zebra, whereas many experiments have been performed in Europe where biting flies are not adapted to striped hosts.

Another challenge for the biting fly hypothesis is why zebra are entirely and consistently striped when various other small black‐and‐white patterns (such as spots, patches or checks) are equally avoided by biting flies. This has been found by multiple authors (Blaho *et al*., 2012; How *et al*., 2020; Caro *et al*., 2023) and yet zebra seem to be under strong adaptive pressure to be uniformly striped with no other form of black‐and‐white patterns on any part of the body.

Regarding the costs of the stripes for this hypothesis, and for ease of comparison, we assume that only one hypothesis is the dominant evolutionary driver: the high conspicuousness of the stripes at short to middle distance would likely increase predator detection and predation risk. All sympatric ungulates at risk from the same predators have adapted relatively more cryptic colouring.

### (2) Thermoregulation

Regarding stripe features, the striped mane and striped belly do not fit the hypothesis. Stripe width is also a poor fit, due to wide stripes on the hindquarters and very narrow stripes on the face. The lack of any other animal with a similar adaptation is also a weakness, as many species live in extreme heat. There is not a satisfactory explanation for why among African ungulates, only zebra are striped. The fact that the hypothesis requires higher temperatures on black stripes is counterintuitive. There is also a lack of scientific evidence to support that stripes can function as a cooling mechanism, Pereszlényi *et al*. (2021) found no evidence for the hypothesised cooling eddies. Also, differential convection above dark and light stripes could only work if the air is not disturbed by a breeze or by the movement of the animal. Zebra constantly move as they forage, and the air around their hindquarters will be disturbed by swishing of their tail. The striping of mountain and Grevy's zebra are a poor fit due to a cooler climate for mountain zebra and thinner striping for Grevy's zebra when an optimum width for striping might be anticipated. However, if there was a functioning cooling effect, the increasing striping vividness gradient from south to north would fit. The greater proportion of white on the hindquarters could help cooling if orientated to the sun: zebra do orient their hindquarters to the sun in the hot afternoon (Caro, 2016), but that benefit would be gained by the white areas and not by the wide black stripes. A greater proportion of white for maximum reflection of sunlight would appear be the coolest in temperature if keeping cool was an important function of the pelage, however, this could have an additional predation cost, as it may be conspicuous at distance. Zebra often rest around noon, similar to many sympatric ruminants, but their foraging activity has not been found to be particularly limited by thermal constraints as they are able to increase their foraging time during the hot late dry season (Owen‐Smith & Goodall, 2014).

The costs of the striped pattern for this hypothesis would include increased conspicuousness at shorter distance with likely increased predation risk and perhaps increased vulnerability to flies due to the short hair that the stripes require.

### (3) Crypsis

**Fig. 2:**
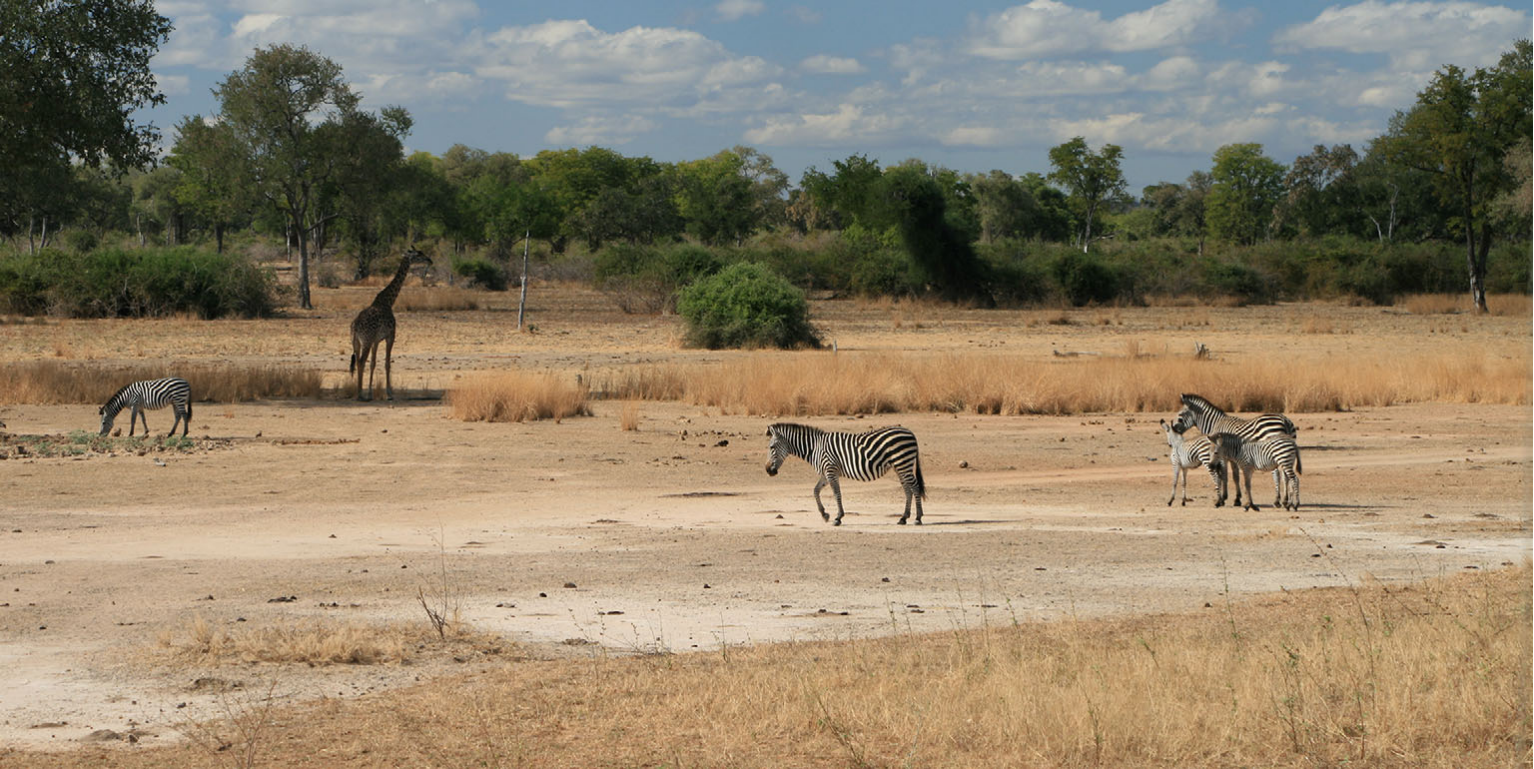
Plains zebra (*Equus quagga crawshayi*) in middle distance showing face and leg stripes not visible and neck stripes only just visible. Rear flank/hindquarter diagonal stripes are the most conspicuous. Sympatric ruminant grazers (and lions) have lower visual acuity in comparison to humans meaning stripes are resolvable only at shorter distances. Photograph by H. Ireland.

The conspicuousness of zebra stripes at short to medium distance negates a crypsis effect at this distance. The mane enlarging the outline and the black line on the mane edge accentuating the zebra outline are counterintuitive for crypsis. Zebra behaviour is also not compatible. However, the pattern could be cryptic at long distance where the grid pattern cannot be resolved and the black and white stripes cancel each other out and merge to an average grey. This would particularly apply to body regions with narrow stripes such as the head and legs (see Fig. 2). Due to tapering black flank stripes and a whiter belly, the average grey shade will pale towards the belly, providing countershading.

Striped patterns can enable a dual function of bold signalling at short distance and crypsis at long distance where the average colour or shade is similar to the background. Similar dual functions of signalling and crypsis using striped patterns have been suggested in reef fish and some invertebrates (Marshall, 2000; Tullberg, Merilaita & Wiklund, 2005; Barnett, Cuthill & Scott‐Samuel, 2017; Berg, Endler & Cheney, 2023).

Costs of the striped pattern for the crypsis hypothesis would include increased conspicuousness at short distance which could increase predation risk at this distance and perhaps increased vulnerability to flies due to the short hair.

### (4) Dazzle colouration

This is still a poorly understood process and is a visual illusion more prominent in two‐dimensional video capture and perhaps less likely relevant for predator vision. For this hypothesis, one might expect stronger striping on rear‐facing parts for confusion of predators chasing fleeing zebra. The striped face seems inconsistent with this. If dazzle colouration was an effective anti‐predation mechanism, the lack of any other mammals employing a similar strategy while under threat from the same predators is a significant weakness. The stripe gradient is not explained. Whether this mechanism could be effective in low light due to reduced total visual input is unclear, but a possibility. However, in low light, the conspicuous white hindquarters might assist predators with visual targeting.

Costs of the stripes for this hypothesis would include conspicuousness at short distance which could increase predation risk and perhaps increased vulnerability to flies due to the short hair.

### (5) Interspecific signalling for mixed‐species herding

The features of zebra stripes appear well adapted for signalling. However, whether or not a signal could help facilitate mixed‐species herding is as yet speculative. However, it is known that plains zebra and multiple sympatric ruminant ungulates prefer mixed‐species herding in the presence of predators or in places or times of danger (Stears *et al*., 2020) and vision is used to find potential herd partners (Beauchamp, 2007). We know that zebra could provide multiple advantages to co‐herding species, are commonly found in mixed‐species herds and form strong associations with other species, particularly with wildebeest (Kiffner *et al*., 2014; Beaudrot *et al*., 2020). However, relatively little is known about the formation, cohesion, integration (relative herd positions) and fission of multi‐species herds.

To form a mixed‐species herd, one species group can join another species group or be joined themselves. If joining another species, there may be a choice regarding which species to join. A potential species to join has to be noticed, identified, chosen and approached. The stripes of a zebra could facilitate these steps. Their eye‐catching pattern is more likely noticed. The pattern is clearly discriminable to ease identification. The striped pattern is a consistent signal to advertise the co‐herding benefits of zebra. Finally, zebra are approachable and non‐aggressive to joining species. When a joining species co‐herd with zebra, the joining species would confer a small predation‐risk reduction and feeding‐efficiency advantage (due to reduced vigilance), on the chosen zebra group. The joining species would also benefit from acting on the signal by reducing their own predation risk while not compromising their feeding efficiency. When zebra join others, the zebra can choose who they join and so the signal seems redundant, however, the signal could function to ease recognition and acceptance, particularly in low light.

A functioning signal could also potentially aid cohesion in moving herds and improve herd integration. If zebra were attractive to heterospecifics, zebra may be able to move and heterospecifics follow. This could have predation advantages for zebra as they prefer to remain mobile at times of increased predation risk, such as at night (Fischhoff *et al*., 2007b; Palmer *et al*., 2017) and could also have feeding advantages. Another advantage of heterospecific attraction would be better integration or a more central position in a mixed herd. A non‐peripheral position in heterospecific herds would lessen predation risk for zebra and allow reduced vigilance. Zebra are often observed to be well integrated in mixed‐species herds, particularly with blue wildebeest.

The striped pattern could have two functions, firstly to gain visual attention so that potential co‐herding species notice zebra, and secondly, as a clear species identifier to advertise the co‐herding advantages of zebra. For the first function, to be noticeable, or demand visual attention, requires a highly conspicuous pattern and the bold black and white stripes are highly conspicuous. Some visual targets are particularly eye‐catching if they harness specific visual attributes that are instinctively noticed. Most published data are derived from humans, which have higher visual acuity but likely similar optical processing. For example, movement is prioritised in visual processing for prey and hunters as it may indicate an animate object (Pratt *et al*., 2010) and also flicker as it is analogous to movement. Contrast changes and edges are also prioritised or enhanced as they may indicate concealed predators or prey or indicate important structures in our immediate environment (Wolfe & Horowitz, 2017). For humans, bold black diagonal stripes (similar to zebra stripes) are one of the most conspicuous patterns (Iizuka *et al*., 2021) and draw visual attention. Black is usually contrasted with yellow as we are trichromatic. As this striped pattern is prioritised in our visual processing, it is eye‐catching and hence used for attracting attention to unexpected hazards. It is not clear why diagonal black stripes are the most conspicuous (a speculation would be to notice overhanging diagonal tree branches). As bold diagonal stripes are so eye‐catching, humans avoid having them on background surfaces, such as wallpaper, as they would be too visually intrusive. For zebra co‐herding ruminants, bold black stripes on a white background are also likely conspicuous and this is supported by black and white stripes on the rumps of impala and Grant's gazelle, likely for following behaviour (Guthrie, 1971). The second function, as a distinctive species identifier, requires a conspicuous, clear and consistent pattern visible from all directions, even when partly visually obscured, and the pattern has to be unique and unlike all non‐zebra species. Zebra stripes appear to be ideally suited to fulfil this function. Heterospecific ruminant ungulates have relatively low‐resolution vision (in comparison to humans) (Melin *et al*., 2016) and the bold stripes can help to allow identification of zebra, particularly in low light.

As a large proportion of predation by lions is at night, it would be important for an interspecies signalling function to operate at night. Zebra and sympatric ruminants (and lions) have excellent night vision due to a higher proportion of rods than cones and large eyeballs with a tapetum lucidum (reflective retinal layer) (Murphy, Hall & Arkins, 2009). They are adapted for low light at the expense of daytime acuity. At night, the white stripes will be visualised against the contrasting black stripes, but the repeating pattern or signal remains the same. The pattern will remain conspicuous in low light at short distance as the white stripes are highly reflective. This will enhance species recognition which may be more difficult in low light. The head and rump have a relatively whiter average colour, the head may help recognition and if zebra are mobile at night the relatively whiter hindquarter could facilitate following behaviour.

If zebra stripes were to act as a signal, could there be any alternative potential recipients? A signal to other zebra to maintain same‐species herds seems unlikely as other herding sympatric herbivores do not require such a pattern and more subtle hindquarter or head markings would likely suffice. An aposematic signal to predators such as lions and hyenas is considered unlikely as the pattern is not localised to a dangerous or defended body part to warn for kicks or bites (Caro, 2016). Additionally, for an aposematic signal to co‐evolve it requires the signaller and receiver to benefit and so a predator should heed the warning and gain an advantage by avoiding the signalling prey (in an environment where there are no mimics). This is not the case as zebra are one of the favoured prey of lions and are also taken by hyenas (Hayward & Kerley, 2005; Trinkel, 2010).

The exceptional species richness and diversity of African herbivores provides widespread opportunities for mixed‐species herding. This species diversity increases moving towards the equator in the north of zebra range and zebra striping intensity appears to follow this gradient (Turpie & Crowe, 1994; Du Toit & Cumming, 1999; Said *et al*., 2003; Larison *et al*., 2015). This would make sense as the relative value of mixed‐species herding to zebra and co‐herding ruminants will increase with herbivore richness and abundance as there will be more opportunity for mixed‐species herding, and historically there would also have been greater numbers of predators due to the higher numbers of prey (Grange & Duncan, 2006; Loveridge *et al*., 2022).

The striped pattern fades to an average grey at distance where the stripes cannot be resolved (Melin *et al*., 2016). This could potentially allow the stripes to fulfil a dual function of increased conspicuousness or signalling at short to medium distance and crypsis at long distance. Long‐distance crypsis could further assist in reducing predation. The stripe width and stripe ratio will dictate the distance where the function would switch from signalling to crypsis. The threshold distance for this functional shift will retract towards zebra at night. The stripe ratio will also dictate the shade of average grey at distance.

The distance from which the stripes can be resolved also differs by body region. The stripes on the rear flank and hindquarters (of plains zebra) are visible from the greatest distance due to wide black stripes and more importantly, wide white stripes (Iizuka *et al*., 2021; Muhl‐Richardson, Parker & Davis, 2021). For zebra vision this would be approximately 140/75/10 m for daylight/dusk/night, possibly slightly less for sympatric ungulate vision and 80/46/11 m for lions (Veilleux & Kirk, 2014; Melin *et al*., 2016). The threshold distance for visualising stripes is slightly greater for sympatric ungulates in comparison to lions, which again could be of benefit to zebra. On the neck, the wide black stripes but thin white stripes mean stripe resolution is limited by resolution of the thin white stripe. This means that the neck stripes are only visible from a shorter distance. It could be that the zebra has adapted the most conspicuous stripes on the rear flank/hindquarters as this region is large in area, relatively high, consistent in position (neck often lower when feeding) and stripes are perpendicular to movement when running. This could allow species recognition from greater distance and enhance following behaviour. The head, neck and leg striping appear best suited to short‐distance species recognition.

Grevy's zebra, and to a lesser extent mountain zebra, have narrower stripes and a higher black to white stripe ratio which would be consistent with a relative greater benefit from crypsis than from interspecific signalling. This is also consistent with their white bellies which could aid crypsis by countershading.

If, conversely, long‐distance crypsis was not of value to zebra and long‐distance signalling was of value, then significantly wider diagonal black stripes (for example 20 cm) with wider white stripes would be better to enable longer distance recognition. However, this could be a disadvantage if it enabled lions to detect zebra and their associating heterospecifics (who often have more cryptic colouration) at greater distance. The thinner stripes of plains zebra appear ideally suited to visually signal at short to medium distance and for crypsis at long distance. In the open non‐complex habitats favoured by zebra, crypsis could likely only be functional at distance.

Criticisms of the interspecies signalling hypothesis would include: why are stripes necessary as multiple sympatric ungulates manage to form large single‐species and mixed‐species herds without stripes? And if zebra needed to identify themselves, why would they need such a striking pattern to do so? However, if more conspicuously striped zebra are more likely noticed and joined by more co‐herding species than less‐conspicuously striped zebra, and if this reduced their predation risk and improved their feeding efficiency, then the more conspicuously striped zebra would be more likely to survive. This could encourage more conspicuously striped zebra over time. Further, if there was no net predation cost for this increased conspicuousness, as paradoxically *via* this conjectured mechanism it could reduce predation at short distance by mixed‐species herding and at long distance by reduced predator detection (due to non‐resolvable stripes), then zebra could evolve to a maximally conspicuous pattern which might help to explain the current phenotype.

Costs of the pattern would be increased conspicuousness to predators at short to middle distance, and therefore increased predation risk, particularly in situations where there was no opportunity for mixed‐species herding. Further there could be increased vulnerability to biting flies.

### (6) Neck striping

As we move south in Africa and zebra striping is less vivid, zebra neck striping remains the most pronounced and persistent of all the pelage striping and is even preserved in the recently extinct quagga subspecies (*Equus quagga quagga*) of plains zebra. This suggests that neck striping may be of the most benefit and more likely evolved first. For interspecies signalling this might fit as the wide zebra neck is a good surface for short to mid‐distance species identification as it is relatively large in area and often elevated in position. Conversely, the strong and persistent neck striping is not such a good fit for the other hypotheses: for the thermoregulation hypothesis we might expect the most persistent striping on the back and upper flanks which face the sun; for the biting fly hypothesis we might expect belly and leg striping to be the most persistent; and for the dazzle hypothesis, the rear pelage should be striped.

### (7) Lion‐predation‐related hypotheses

In many articles regarding zebra stripes, avoiding lion predation utilising any mechanism is dismissed or excluded as a potential driver of striping because lions kill a significantly greater proportion of zebra than expected for zebra abundance (Hayward & Kerley, 2005; Caro *et al*., 2014; How *et al*., 2020). This is misleading. Lions specialise in hunting larger prey, like zebra, and a large proportion of zebra mortality is due to lions. In a predator/prey evolutionary arms race where lions are adapted to specialise in killing large prey such as zebra, zebra are likely adapted to reduce lion predation. For example, if we look at another zebra attribute, acceleration speed (which incidentally is greater than that of a wildebeest; Elliott *et al*., 1977), we would not conclude that this speed is unrelated to lion predation because lions take a greater proportion of zebra relative to their abundance. Further, if we look at the prey species within lions' preferred weight range, we find lions actually kill slightly more wildebeest despite zebra being heavier and closer to a lion's ideal prey weight (Hayward & Kerley, 2005). As a relatively large proportion of zebra mortality is due to predation (lion and hyena) (Grange *et al*., 2004), any adaption that influences this even slightly would be of relatively large benefit to the survival of an individual zebra. Therefore, it is inappropriate to exclude predation as a driver for zebra stripes, and further, as zebra are under such high selection pressure from predation, it remains feasible or likely that the driver for zebra stripes is related to predation. Furthermore, as the stripes increase the conspicuousness of zebra, which is counterintuitive in avoiding predation, and as predation has been shown to be the most important factor controlling zebra survival, it appears more likely the stripes have a net beneficial effect related to predation to explain their strong adaptation.

Additionally, for the biting fly hypothesis, a hypothesis not directly related to predation, if zebra were adversely affected by a fly‐carried disease, this would make them more vulnerable to predation, meaning adaptations to protect against predation would again be of value.

### (8) Combinations of hypotheses

A combination of functioning hypotheses is possible. For example, the stripes could have different functions at different distances from the observer. At long distance, the stripes could average to grey to function for crypsis to reduce predation risk for zebra. However, as this does not require stripes this would be a secondary function and not the driver of stripes. A combination of interspecies signalling and long‐distance crypsis could allow refined stripe‐width selection to set the threshold distance where the value of one function switches from one to another. Deterrence of biting flies which has been shown to work at short distance could also be possible in combination with long‐distance crypsis. Or a triple function of biting fly deterrence at shortest distances, signalling at short to middle distance and long‐distance crypsis would also be feasible.

## VIII. CONCLUSIONS

(1) A problem for all these hypotheses is their novelty. Stripes to avert biting flies and stripes for thermoregulation have not been described in any other species. Stripes for dazzle colouration have not been described in any mammals or birds. Stripes for long‐distance crypsis have been described in fish and invertebrates but not in mammals. Stripes to facilitate mixed‐species grouping have not been described in mammals. For all these hypotheses, the lack of alternative example species deprives us of comparative research, and also provides a need for a satisfactory explanation for why only zebra are striped.
(2) The biting fly hypothesis is the only hypothesis with direct supporting evidence (albeit mainly from Europe). However, the hypothesis and supporting arguments have some potentially important unresolved elements: inconsistent stripe features like the invulnerable striped mane, non‐striped belly and legs, highly variable stripe width and the fact that alternative non‐striped black‐and‐white patterns seem equally effective. There is also a lack of a cogent reason for why only zebra utilise this defence; the effects of fly‐carried diseases do not appear likely to be an extra burden for zebra compared to sympatric herbivores and the short hair argument does not seem satisfactory. Furthermore, zebra appear equally bothered by flies compared to sympatric species. The stripes may help to deter some biting flies but these inconsistencies reduce our confidence that this is the only or main driver of the stripes.
(3) The thermoregulation hypothesis has incompatibilities with stripe features such as the mane, belly and stripe width; it has a lack of supporting evidence and the underlying mechanism seems less likely. There is no satisfactory explanation for why only zebra are striped. Overall, we suggest that this hypothesis appears less worthy of further investigation.
(4) The dazzle hypothesis has multiple incompatibilities with various stripe features, the mechanism is poorly understood but possibly could function in low light; however, this also seems less likely as if it did function as an effective antipredator device, sympatric mammals in danger from the same predators would inevitably mimic the pattern over time. Again, we would suggest that this hypothesis appears less worthy of further investigation.
(5) The crypsis hypothesis is unlikely at short distance due to increased conspicuousness but could function at longer distance where the stripes are averaged to a shade or luminance similar to background, also with countershading. However, this cryptic long‐distance effect could be achieved with an averaged grey shade and without the stripes and so this would be an additional long‐distance function and a separate hypothesis is required to explain the stripes.
(6) The signalling for mixed‐species herding hypothesis is speculative but is consistent with zebra stripe features and the stripes appear well adapted for signalling at short to medium distance. There is evidence showing the benefits zebra could provide to co‐herding ruminants, and hindgut digestion combined with foraging ability could possibly provide an explanation for why striping occurs in zebras but not in co‐herding species. The exceptional species richness of African herbivores providing widespread opportunities for mixed‐species herding combined with the hindgut physiology of equids could allow a unique co‐herding niche that does not exist in other continents. However, importantly, there is currently no direct evidence to support zebra stripes having a signalling function.
(7) From logical argument and empirical evidence, we suggest that of all the theories discussed in the last 25 years, it is the biting‐fly‐protection and interspecific‐signalling hypotheses that seem most appropriate for further study. In addition, long‐distance crypsis may be a secondary function in combination with another hypothesis. For the biting fly hypothesis, further evidence for zebra stripe deterrence of East African tabanids or other East African biting flies would be valuable. Evidence for the costs to zebra from biting flies, or from diseases carried by biting flies, in comparison to other large grazers in the same environment would be very useful. For interspecific signalling, more evidence regarding the formation, fission, cohesion and relative herd positions of mixed‐species herds containing zebra would be required, both in the dry and wet seasons and ideally during the day and night. Cameras (ideally night vision) mounted on zebra collars might have the potential to assess associating species. Drones could also be of value. Evidence to assess whether zebra alter their foraging behaviour to accommodate ruminants would also be very helpful. More direct evidence for a signalling function of stripes could be obtained by assessing whether corralled zebra or dummy zebra are more attractive to ruminants than control ruminants or horses (as surrogates for unstriped zebra). For long‐distance crypsis, further estimation of the distances where stripes can be resolved by lions and by the ruminant species that herd with zebra would be useful.
(8) Ultimately, the research does not yet show why zebra stripes were selected and maintained. To answer this longstanding question, more work needs to be done and we should remain careful before declaring the question resolved.

## AUTHOR CONTRIBUTIONS

H. M. I. conceptualised this review and wrote the first draft. H. M. I. wrote subsequent drafts with contributions from G. D. R. H. M. I. and G. D. R. edited the final draft and approved the final version.
